# The experiences of the caring dyad: (Un)articulated realities of living with cardiometabolic risk, metabolic syndrome and related diseases in severe mental illness

**DOI:** 10.1111/hex.13322

**Published:** 2021-08-02

**Authors:** Dolly Sud, Ian Maidment, Eleanor Bradley, Jonathan Tritter

**Affiliations:** ^1^ Aston Pharmacy School, College of Life and Health Sciences Aston University Birmingham UK; ^2^ Pharmacy Department, Leicestershire Partnership NHS Trust, Bradgate Mental Health Site Glenfield Hospital Site Leicester UK; ^3^ College of Life, Health and Environmental Sciences University of Worcester Grove UK; ^4^ School of Humanities and Social Sciences Aston University Birmingham UK

**Keywords:** dyad, informal carer, patient, qualitative, relationship, schizophrenia, thematic analysis

## Abstract

**Background:**

Informal carers play an important role in the care of patients with mental illness. Little is known of the relationship experience of the patient and their informal carer (caring dyad) as the context for the intersection between physical and mental health.

**Aim:**

This study aimed to explore the impact of comorbid cardiometabolic risk (CMR), metabolic syndrome (MetS) and related diseases and severe mental illness (SMI) on the caring dyad.

**Design:**

Between October 2018 and March 2020, we conducted 11 in‐depth semi‐structured interviews across 6 adult caring dyads, interviewing each individual separately.

**Setting:**

Dyads were recruited within the United Kingdom; informal carers were nominated by the patient as a person who provided a significant amount of support.

**Variable Being Studied:**

Participants were asked about the impacts of illness and caring on daily life.

**Data Analysis:**

Data were analysed at the dyad level using thematic analysis, comparing and contrasting responses from each individual.

**Results:**

Themes were identified: enhanced closeness, dissonance and balance within the caring dyad.

**Discussion and Conclusions:**

This study uses a particular population of patients with comorbid CMR factors, MetS and related diseases and SMI and their informal carers to explore the relevance and utility of caring dyads as an analytical framework to inform practice and policy. Future interventions should consider factors impacting on dyadic relationships to formulate effective and sustainable dyadic care and treatment to improve health outcomes for both patients with SMI and their informal carers.

**Patient/Public Involvement:**

In this study, patients and informal carers were participants. Topic guides were piloted with a patient and informal carer.

## 1 INTRODUCTION

Informal carers play a vital role in supporting patients with severe mental illness (SMI)^1^ and are an important social contact for patients who experience high levels of social exclusion, isolation, loneliness and stigma. Informal carers may be the first to notice signs of relapse in mental health^1^, ^2^ and often help patients engage with treatment.^3^, ^4^ Quality of life may be improved in patients with support from informal carers;^5^, ^6^ they play a fundamental role in advocating and engaging service input and support.^7^ Support from carers is associated with significantly reduced rates and duration of psychiatric hospital admissions^8^ and is acknowledged in relevant guidelines.^9^, ^10^, ^11^, ^12^, ^13^

Patients with SMI have poorer physical health and reduced life expectancy (approximately 15–20 years) compared with the general population.^14^ The majority of deaths in patients with SMI are due to preventable physical diseases, in particular, cardiovascular disease (CVD); they have a 2–3 times higher risk of dying from CVD compared to the general population.^14^ This mortality gap exists in countries considered to have high standards of healthcare^15^ and can in part be accounted for by a higher relative risk^16^ for modifiable cardiometabolic risk (CMR) factors, metabolic syndrome (MetS) and related diseases.

Informal carers typically play an essential role in supporting patients to manage their illness and CMR, MetS and related diseases, contributing to patients' healthier lifestyles by, for instance, advice about food choices or preparing meals.^17^ They also support attendance at health appointments and participation in physical activities.^17^ This study focused on a sample of patients with comorbid SMI and CMR, MetS and related disease and the nature and consequences on their lives and illness of the involvement of their informal carers.

The aim of this study was to explore the impact of comorbid CMR, MetS and related diseases and SMI on the caring dyad. The objectives are to^1^ explore the role of the caring dyad and^2^ explore the utility of the caring dyad as the unit of analysis (as opposed to individual experiences) in this space.

Sustainable lifestyle modification and pharmacological treatments are needed to address CMR, MetS and related diseases^14^ and typically rely on informal carer participation.^17^, ^18^ We argue that to understand the complex interactions that underpin the management of long‐term comorbid conditions, it is necessary to situate the experience of patients within the context of the caring dyad. Similarly, the experience of illness is understood far better as a product of interaction within a dyadic relationship. Comparing and contrasting views of everyone within the dyad generate knowledge of the coconstruction of illness management within the dyad and the limitations of clinical engagement solely with a patient.

### 1.1 Caring dyad

Our analysis draws on the interdependence theory^19^, ^20^, ^21^ to understand CMR, MetS and related diseases and SMI and their link with closeness, dissonance and balance within the caring dyad. This theory considers the ways in which bilateral influence within the dyad affects the outcomes (behaviour or experience) of individuals. The dyad provides a critical unit for the analysis of health behaviours^22^ where the characteristics (beliefs, identity and actions) of the interacting partners affect the outcomes of one or both individuals.^20^ We argue that the experience of CMR, MetS and related diseases and SMI is a dyadic phenomenon. The management of illness has consequences for the dyad and can lead to enhanced closeness as well as dissonance in the relationship and inevitably shapes both individual and collective identity and joint experiences of illness.

Although less well explored, self‐regulation in research on dyads or carers^23^ explicates the processes by which individuals become aware and respond to health threats.^24^ We propose that CMR, MetS and related diseases and SMI are a shared health threat^25^ for both individuals within a dyad. Further, the dyad has collective responsibility and is a key unit for illness management. However, as an interdependent team, relationship‐level factors such as communication and relationship quality can affect the ability of the caring dyad to manage illness constructively and collaboratively. Our study explores the challenges to the dyad as a functional social unit from the perspective of both the patient and the carer. In particular, we consider decisions by carers to compromise their own needs, role conflict and symmetry/asymmetry in the provision of care within the dyadic relationship.

This study provides an important element missing from existing understanding: The interaction between dyadic relationships and health outcomes. Our methodological approach lends primacy to understanding the relationship dynamics within the caring dyad as a way of understanding the consequences for illness management. Further, we conceptualise these dynamics as the main route to dyadic health and functionality within the context of illness; the presence of CMR, MetS or related diseases and SMI can generate both enhanced closeness and dissonance within dyads as well as challenging role identities and balance within the relationship, which in turn affects health outcomes. Our analysis focuses on how CMR, MetS and related diseases and SMI interact with, and impact on, the relationship within the caring dyad and correspondingly on illness management.

## 2 METHODS

### 2.1 Sample and recruitment

Between October 2018 and March 2020, we conducted 11 individual in‐depth semi‐structured interviews constituting six caring dyads; one patient participant had two carers and was therefore part of two dyads. The dyad consisted of a patient with SMI and CMR, MetS or related disease and their informal carer. Informal carers' ages ranged from 26 to 69 years, and patients' ages ranged from 43 to 64 years. All participants identified themselves as being from a white ethnic group and further details were not sought. Individuals from the United Kingdom were recruited via posters placed in clinics and patient areas within one mental health NHS trust, patient and public involvement websites, mental health websites and social media (Twitter).

Patients were eligible to take part if they were (i) ≥18 years, (ii) had a psychiatric service diagnosis of an SMI (schizophrenia, bipolar affective disorder, schizoaffective disorder or any other nonorganic psychoses) and (iii) had a confirmed diagnosis of CMR, MetS or related condition (smoking, obesity, overweight, abdominal obesity, lipid abnormalities or specific treatment for lipid abnormalities, hyperglycaemia, prediabetes, type 2 diabetes, hypertension or treatment for hypertension, insulin resistance and MetS).

Informal carers were recruited through the patients participating in the study; patients were asked to nominate a noncare professional (≥18 years) who provided them with a significant amount of support. A study information pack (containing a letter of invitation and participant information sheet) was provided to the patient to give to their nominated carer. The lead researcher checked the eligibility criteria of the participating carers.

After enrolment, each individual was asked to provide informed consent, was reimbursed for out‐of‐pocket and travel expenses and was offered a £10 gift voucher as recognition for study participation. All participants with CMR, MetS and related diseases and SMI recruited to the study referred to themselves as patients; therefore, we adopt this term. Before the interview, demographic and clinical data were collected.

This study received ethical approval from the West Midlands—Coventry & Warwickshire Research Ethics Committee (REC reference 18/WM/0291), the Health Research Authority, Aston University and the Leicestershire Partnership NHS Trust Research and Development Department.

### 2.2 Data collection

Semi‐structured telephone interviews^26^ were conducted by the lead researcher, a senior mental health pharmacist (D. S). The topic guide was informed by a literature review,^27^ clinical background and experience of the lead researcher and pilot interviews with a patient and an informal carer. The literature review identified a lack of qualitative research exploring the dyadic view of CMR, MetS and related diseases and SMI. Open‐ended questions explored the impact of CMR, MetS and related diseases and SMI on daily life. Separate parallel topic guides were devised for participating patients (Appendix 1) and carers (Appendix 2). Probes, incorporated into the topic guides, were used to prompt further and fuller responses. Details of the relationships within each dyad are provided in Table 1—all information is reported verbatim. Identifiable information was changed to ensure anonymity. Participants were located in their own homes and on their own at the time the interview took place.

**Table 1:** Key characteristics of dyads (please note that all information is self‐reported verbatim)

Dyad	Participant code and relationship	Gender	Live together
1st Dyad P1 and C1	P1	Female	No
	C1 (sister of P1)	Female	
2nd Dyad P2 and C2	P2	Female	No
	C2a (romantic partner of P2)	Male	
3rd Dyad P3 and C3	P2	Female	No
	C2b (brother of P2)	Male	
4th Dyad P4 and C4	P4	Female	Yes
	C4 (husband of P4)	Male	
5th Dyad P5 and C5	P5	Female	Yes
	C5 (husband of P5)	Male	
6th Dyad P6 and C6	P6	Female	No
	C6 (daughter of P6)	Female	

*Note:* P is a patient with cardiometabolic risk, metabolic syndrome or related diseases and severe mental illness; C is an informal carer of P.

All information is self‐reported verbatim.

### 2.3 Data analysis

Interviews were expected to last from 45 to 60 min (Appendix 1). The actual interview times ranged from 24 to 94 min, were audio‐recorded and transcribed verbatim. The lead researcher listened to each recording twice whilst simultaneously reading the transcripts. Transcripts were uploaded into NVivo version 12.^28^ Analysis was carried out with and without NVivo using thematic analysis (TA).^29^ TA uses both inductive and deductive reasoning to analyse data.^30^ Framework analysis, a type of codebook approach,^30^ was considered a better choice than the reflexive TA approach as it emphasises both a priori issues and themes identified from the data to guide the development of the analytical framework. This approach suited the aims of this study in that there were predefined areas for exploration but also a need to be open to discover the unexpected. Framework analysis has five main stages.^31^, ^32^, ^33^

The lead researcher (D. S.) carried out the data analysis by comparing and contrasting the transcripts of each individual within dyads^34^ to discern similarities and differences and identify patterned meanings.^34^, ^35^, ^36^ A reflexive journal and field note diary were also used to inform analysis. Regular discussion with co‐authors took place throughout the analysis.

## 3 FINDINGS/RESULTS

The analysis identified three key themes: Enhancing closeness, dissonance in the dyad and creating balance within the dyad. The presence of CMR, MetS and related diseases and SMI had a major impact on the relationship between patients and their informal carers. Individuals within a dyad found ways of negotiating a balance that enabled and reinforced successful illness management for both parties; this was linked to a recognition that carers were also patients. Engaging in mundane activities related to illness management including physical activity, attending appointments and informal carers meeting the unmet needs of the patient enhanced the closeness of the individuals within the dyad. Dissonance within the dyad was associated with the patient ignoring advice from their carer, informal carers participating in controlling and bullying behaviour and incongruent beliefs about health and well‐being. Within the caring dyad roles, identity and balance were affected by how needs were framed, met or compromised.

### 3.1 Enhanced closeness with the caring dyad

A significant proportion of the dyads reported enhanced relationship closeness as a result of collectively implementing lifestyle changes. This generated a sense of ‘team’, ‘togetherness’ and openness; together, they collaborated in managing CMR, MetS and related diseases. Lifestyle changes varied, but many respondents referred to physical activity. Similarly, the joint engagement with formal services was a central element that created the greater closeness reported by some dyads.

For both patients and carers, undertaking regular physical activity, such as walking together, was regarded as an essential part of the support enabled by and through the dyad.

I have great support from my brother. Like yesterday, he came around at quarter past six in the morning and made me go for a walk. Which is great. (P2)

She does enjoy walking a lot and we try and go out and walk together every day, that's what keeps her agile and mobile. Her health is very good, considering the challenges. (C2b)

A significant proportion of dyads reported that attending therapy together fostered a greater level of understanding and that this led to a sense of mutual support and closeness.

…we are all having family therapy at the moment, we've been learning about the triggers, what things lead me to become ill, and that's been really useful to share with the family, so everybody's aware when things are starting to deteriorate. It has brought us closer together. (P2)

The informal carer of this patient also saw the benefit for everyone directly involved:

…she's been given family therapy to help with her relationships with her dad and to help us sort out her social networks so that the family, her partner, my wife, me, we can understand her health better. (C2b)

Paradoxically, the experience of receiving little or no external support for their conditions reinforced togetherness. A carer explained that providing support to his sister that was not available from healthcare services created a space for him to play a greater role, increased his understanding and enhanced their closeness.

We work together a lot but she's not receiving any outside help at all, I don't think, from the system which I think is a shame. I work with her a lot on her diet and we talk a lot. Since our mum passed away, she's started cooking for dad and I've been helping her to do that and so it's kind of like doing a cooking course for her and that's been really helpful for her to learn and become confident about how food is made and how that impacts on her health. (C2b)

When asked if there had been any formal diet and lifestyle advice, this informal carer said,

I think they're up for it but I think that the help and support that she's (the patient) been given haven't been that helpful because we already know what we are doing (C5)

Undertaking these lifestyle activities together, as a dyad, often improved the health of both the carer and the patient. In addition to these reciprocal positive impacts, it also served to insulate the patient from the inadequacies of healthcare provision.

Participating in everyday mundane activities, as well as those specifically associated with the illness, strengthened relationships. In many ways, mundane activities were also essential aspects of disease management. For instance, using a fitness app together facilitated reciprocal encouragement.

…It was my daughter that said, ‘well why don't you try My Fitness Pal’ and I said ‘right, I'll do it’ and we did it together. I just stuck with it, I didn't eat anything other than my meals and I went walking every day=… the weight just dropped off. ……the app has been fantastic and doing that sort of thing with somebody else is great because you can boost each other… (P6)

A sense of a joint endeavour often predated the emergence of the caring relationship.

…with regards to weight, health, eating healthily and living healthily, me and my mum are very open, we used to go to the gym together when I was younger, as a team. (C6)

In addition, joint activities could be revisited as a source of affirmation, reinforcing closeness and reciprocal support.

This last couple of weeks we've both gone back onto it, just eating what we've planned for meals and no biscuits in‐between or anything like that, no alcohol or no takeaways or anything. (P6)

### 3.2 Dissonance within the caring dyad

While some responses to the illness trajectory generated closeness within dyads, there were three primary sources of dissonance: Incongruence in expectations and beliefs about health, well‐being and quality of life, barriers or ‘shutting out’ a carer's attempts to provide care and controlling or coercive behaviour.

Two particular beliefs generated conflict: The prioritisation of mental over physical health by some informal carers and differences in what constituted health, well‐being and quality of life. All patients confirmed that mental and physical health were equally important to them. However, for their carers, this was not always the case. Some carers reported that mental health took prominence.

To me, my physical health is no less important than my mental health. My mental health is just as important. They are so intertwined with each other. When I'm mentally not well, then I'm physically not well and vice versa…for example, if I stop exercising or stop eating properly my mental health gets worse. (P2)

I would say you've got to sort the mental health because I think you've got to be calm and stable, as stable as you can be, before you can address physical health which is a side issue. …the mental health is more important, it drastically affects the lifestyle and health decisions, like, what kind of food she (P4) might choose. (C4)

Importantly, the impact of the side effects of medication, an aspect of physical health, was also viewed differently within dyads. Patients focused on the aesthetic consequences and how it made them feel about themselves in terms of self‐esteem and confidence. Whilst acknowledged and appreciating these impacts, informal carers were far more concerned about the long‐term physical health consequences of conditions such as diabetes or heart disease.

When I had my first crisis of psychotic depression, I was very thin, I was a size six. When I started taking medication, I put weight on initially gradually, now I've got to about a size 14. I don't feel very comfortable with it. For a very long time now I have been overweight, I haven't got used to it, I feel like, like, I don't like myself and I feel not very confident and very upset it's hard to describe. I lack self‐esteem and don't feel like getting dressed nicely sometimes. I need to buy the largest clothes. (P4)

The husband of this patient was worried about the impact of long‐term physical health conditions and how engaging with these were part of his responsibility.

Yeah, naturally it's (the weight gain)…I do think there's an element of just self‐consciousness and being uncomfortable for her. But for me it's a concern because, having worked in hospital, I'm all too familiar with how prevalent diabetes is…it's naturally a consideration and something that concerns me because it would only add to my own caring responsibilities. (C4)

For some individuals, side‐effects related directly to self‐esteem.

 You just feel so bad about yourself, your self‐esteem really does plummet with the weight gain. (P2)

However, for her brother, the concern was not only about the physical health consequences but also the potential implications for mortality.

So, the diabetes, cardiovascular health and the weight gain causing shortening of life are my principal concerns. But, you know, my sister, well, we all have our vanities and I'm sure she would like to look slender and like the models but the thing that I think is the threat of long‐term illness from it. (C2b)

The prioritisation of health issues differed between patients and their informal carers; carers adopted a longer‐term view of physical health, while those they cared for were more concerned with the impact on day‐to‐day activities and issues of self‐esteem and self‐confidence.

Informal carers appeared to project their beliefs onto the person they cared for, creating challenges when these beliefs differed. One patient described what health, well‐being and quality of life meant to her.

For me it would be contributing to my community and to the society that I live in…. I would be working and I would be paying tax that provides the services that people need in my community and my society. (P5)

This account was very different from the understanding of her husband.

She's had a down patch, which I think she's coming out of…… I know she finds it difficult because she can't just jump back into work, anyway me and the consultant won't let her, and rightly so, but you know that does impact, because she wants to feel as though she's contributing to society ….and (me) saying (to her –P5) well, you are actually contributing, you're contributing in this way rather than this way. But do you have to contribute to society? (C5)

Such contrasts in the understanding of the situation and the implications that this had for appropriate responses were a source of tension within dyads.

Informal carers discussed how their advice, often repeated, was not heeded, for example, explaining food labelling to help make healthy choices or the impact of being overweight.

…she just didn't get it at all, and the number of times I've tried to explain things to her she just, she just shuts down, she can't process it, she can't understand. (C6)

I say to her: ‘Well, what do you know, does that not bother you?’ because it would bother me, and she says, ‘I know, I know, yeah’, so I hit that lovely polite brick wall. (C1)

The informal carers felt that this often resulted in the person they cared for withdrawing. Sometimes, this led to a harsh response by the carer or the use of bullying that might be understood as coercive or controlling.

For one patient, their informal carer sought to impede food consumption at night while another used shame as a tactic.

….she comes down during the night and she eats lots of food …. I lock the (kitchen) door at night on a regular basis. (C2b)

And sometimes my sister will take a photo of me to show me how I look and how round I am around the middle. She took one yesterday and said, ‘Look, here, this is what you look like, just look. You know, I'm concerned about just how much weight you've put on, aren't you concerned?’ (P1)

Bullying often took the form of projecting the impact of morbidity as opposed to death and often reflected the incongruence in beliefs and visions of life between the patient and the informal carer.

I try and shock her as much as I can, because I say, ‘Well, you know, if you have a heart attack and die, that's fine. If you have a stroke, then you're looking at a different life forever’. (C1)

I feel the weight gain has impacted on her quite lot but it doesn't seem to bother her, she just is living with the fact of. If we go out for a walk, she'll look for a bench early on, she's breathless, and she doesn't walk as far. I know she's aware she's breathless, and, you know I am cruel. I'm very open with her, and I'll say, ‘Hey kiddo! You know, our mother at 88 wasn't like this, you're just 64 and you already have so many physical limitations’. (C1)

Typically, such tactics had a negative impact on the patient.

Well, it just makes you feel a bit down and, you know, a bit told off. You know these things but you know she's doing it out of concern, because she's concerned…Being told off about it too much stops me from doing things. (P1)

### 3.3 Balance within the caring dyad

What each individual within the caring dyad wanted for themselves, as an individual, was different from how they framed their needs within the context of the dyad. Many carers had their own health needs, but explicitly conceptualised their identity as someone caring for another rather than someone with a long‐term health condition. For some carers, the dyad created an opportunity to redefine their identity but also to negotiate greater symmetry within the relationship. Being a ‘partner’ in care moderated a focus on their own health concerns.

I paid for a private scan it came up with a chronic fractured and severe stenosis spine of the spine and torn ruptured discs. Now I've got a heart problem, I've also got an autistic son who lets us give him massages and, hug him. When I go home, back home to her (P2) house I get all of her and sometimes, not very often, I just think it's too much, but then I just think how lucky I am, it's my job to take care of her you know, a few times in the past she said you know, she couldn't live without us. (C2)

For some informal carers, however, their responsibilities within the dyad led to compromising their own needs whether health related or in their personal or professional life. Their role as an informal carer took primacy as this carer describes in relation to a consultation with his GP.

I'm suffering from chronic and acute stress, the GP said not a great deal can be done really unless I just pull myself out of the situation and have a three month holiday. I think on the positive side, I'm quite lucky because I've got no health illnesses but I'm pushed to the point of exhaustion really, where I could barely eat, like I couldn't eat until about two or three in the afternoon. I've just got to keep plodding on really and just try to ration the effort I put in. (C4)

One carer recalls her mother finding things particularly difficult in the weeks preceding her wedding.

A few weeks before my wedding she (my mum) had a wobble, she started kicking off about things It's almost like she needs to know that even though something's big going on with me I'm still going to be there for her. I put up with it because she's my mum and I love her. (C6)

There were also examples of dyads where care was more symmetrical; the patient also provided care for their informal carer. This finding challenges the notion that the provision of care within dyads is entirely unidirectional. Reciprocity of care suggests greater equality within the dyadic relationship, an overlapping of the roles and degree of symmetry of work, particularly in terms of emotional labour. This was especially apparent when respondents were asked what was important in their everyday life.

Oh, when I'm able to go to work to do my shift that I need to do and meet people and be able to socialise to talk to them and feel happy and fit. I also love spending time with my children looking after them and looking after the house and my husband. Yes, my husband is my carer as well. These are the times when I'm quite happy and content with life. (P4)

…so, the best quality of life I can possibly think of, being realistic, is that I continue as if everything is good and stable. Things like looking after my dogs and walking them. Also, looking after my husband, even though he's my carer. (P5)

Greater symmetry in roles and caring labour within the dyad was more apparent when carers had a long‐term condition.

We know what we're already doing [in relation to long‐term conditions], I've got diagnosed with diabetes and we did all of that then, changed our lifestyle, so it's healthier, exercising, eating as healthy as we can. We've cut down on, processed food and stuff, we eat a lot more fruit and veg than we used to. (C5)

## 4 DISCUSSION

This article addresses the absence of literature that utilises dyad as the unit of analysis for comorbid physical and mental illness. The impact of CMR, MetS and related diseases and SMI on caring dyads varied, but had consequences for illness management, identity and the quality of the relationship. For some, being in a dyad enhanced closeness; they were able to work together as a team. This was particularly apparent in more symmetrical caring relationships where both individuals within the caring dyad had health needs and support was reciprocal. Jointly pursuing activities associated with well‐being also created a sense of individual and collective agency. For others, the impact was negative, leading to carers being controlling and bullying and the patient shutting their informal carer out. The different roles within dyads and their dynamic nature had consequences not only for the way individuals lived and managed their conditions but also on their identity and how they negotiated balance within the dyad.

Relationship quality within the caring dyad played a key role in illness management. Those dyads in which respondents took part jointly to implement lifestyle changes reported enhanced closeness. This is consistent with previous research on a programme to increase social support for healthy eating and exercise that reported benefits to relationship quality.^37^ Dyads in our research attributed the improvements to spending more time together and learning more about each other's experiences and perspectives. These results are also supported by the theory of dyadic illness management; dyads with a better relationship quality may be better equipped to work together to manage health behaviours.^38^ Our findings also show that taking part in mundane tasks contributes to well‐being but also to a sense of agency for the patient and their informal carer.

We suggest the need for a new theoretical framework to better understand the management of SMI and comorbidity that incorporates the dyadic interdependence theory and the dyadic illness management theory and the common‐sense theory of self‐regulation. This framework needs to take account of potential negative behaviours and the health consequences of controlling and coercive action and the impact of incongruence in beliefs about illness.

These findings provide a starting point for further research into dyadic illness management within the context of comorbid physical and mental illness. In addition, we provide evidence for formulating more effective interventions targeting dyads rather than solely patients. Such interventions will need to engage with both individuals and address relationship quality, promote dyadic implementation of lifestyle changes and identification and reduction of factors that lead to controlling and coercive behaviours.

## 5 IMPLICATIONS FOR PRACTICE

While further research is needed, this study provides evidence that supports the need for more formal recognition of the active role that informal carers play in supporting CMR, MetS and related diseases in SMI. The findings suggest that supporting informal carers may improve patient care.

Informal carers are vulnerable because of their caring roles and the consequence of the informal carer not functioning can result in dissonance in the relationship and loss of support within the caring dyad. Again, interventions focused on the informal carer can maximise benefits to both the patient and the informal carer and help ensure greater symmetry and balance within the dyad.

The nature of the support for carers needs to consider the management of tensions (e.g., bullying) within dyads. Support for the informal carer will, in part, be about helping them manage not to bully and recognise that their caring role can lead to compromising their own healthcare needs.

Focusing on the informal carer has the potential to facilitate a greater longevity in their role. Viewing the dyad, rather than the individual patient as the object of formal care, recognises that a key target of an intervention should not be the patient and their needs but also the carer and their needs. Focusing on the dyad allows us to better articulate treatment regimens that have the potential to facilitate improved outcomes for both the informal carer and the patient.

Given the substantial and valued contribution that informal carers make to patient outcomes and the negative impact on their own health,^17^, ^18^, ^39^, ^40^ offering support to informal carers in SMI has been included in several treatment publications.^41^ However, recognition of the importance of the interdependent relationship between patients and informal carers and the overlap between their caring needs as a dyad is absent.

## 6 IMPLICATIONS FOR RESEARCH

Patient outcomes need to be understood in terms of the caring dyad as they play a significant role in mediating, mitigating and supporting treatment. Future research should consider the dyad as a way of understanding how people with long‐term conditions engage with healthcare and learn to live with their condition. Research focusing on compliance and recovery, for instance, could usefully consider the part played by informal carers in facilitating or limiting such processes. Ensuring that the informal carer is conceptualised as an active agent within the caring and curing process would more fully recognise this essential element of the lives of most people with mental illness and long‐term conditions.

In addition, evidence presented here also demonstrates that informal carers place primacy on mental health over physical health that reinforces clinical practice. Long‐term illness management is a challenge for healthcare systems that are mainly organised to deal with acute episodic care, rather than long‐term conditions.^42^, ^43^, ^44^, ^45^ Such considerations also echo behavioural interventions that focus on learning to live with illness rather than surviving it.^46^ Further research should build on the initial findings here: That the dyad as a unit of analysis reinforces the importance that informal carers place on mental health but also highlights their different interpretation of physical health issues in SMI such as CMR, MetS and related diseases and, further, that long‐term management is about behavioural modification but also about the long‐term consequences of the side effects of psychotropic medication.

Finally, holistic care is more than simply considering together the individual aspects of physical and mental illness but rather to understand the consequence of their coexistence and how this can be managed. Future research should focus on the use of dyads to facilitate understanding this better as an integral aspect of lived experience.

## 7 LIMITATIONS

Participants' experiences may not represent those from ethnic groups other than white British, other genders than those who identify as female or those who reside outside of the United Kingdom. For example, there were no respondents with a Chinese ethnic background. None of the patients recruited into the study were currently being treated as a psychiatric inpatient. However, it is acknowledged that such patients may have been too unwell to consent.

Qualitative research is concerned with assessing validity through the richness of the data collected rather than the number of participants and generalisability. However, given the groups not represented, the results and findings might not be generalisable to those not represented. A greater breadth of views and richer data may have been collected if a more diverse set of individuals had participated in the research.

We did not collect any data about the severity or duration of mental or physical illness or medication use, which might have a consequence on the way dyads functioned.

All demographic and health information was reported verbatim; it is possible that these data may lack accuracy. A number of studies have found no consistent relationship between self‐report accuracy and demographic factors, such as gender, age and health status.^47^ Older age is the only factor that has been identified to be significantly associated with a lack of accuracy and under‐reporting of healthcare utilisation.^47^

All interviews were conducted by telephone and we acknowledge the absence of communication that face‐to‐face interviews provides. Our interview method may have limited rapport between the researcher and the participant. However, studies suggest that the quality of data collected from face‐to‐face and telephone interviews may be comparable.^26^ Interviewing vulnerable individuals such as patients with SMI can be seen as challenging; careful planning can maximise the opportunities to gather in‐depth qualitative information.^48^ As recommended in the literature,^48^ the lead researcher took steps to ensure that the researcher–participant relationship created a safe space for the disclosure of sensitive information and allowed patients to tell their stories. Interviews with each individual within the dyad took place at separate points in time and in no particular order. We accept that responses may have been affected by potential discussion between individuals within the dyad between individual interviews.

Recruitment of informal carers via the patient may have limited recruitment and can be associated with a number of challenges.^49^ Patients may be reluctant to acknowledge the need for informal carers or may not think of family members or persons helping them as informal carers. Informal carers may not see themselves in this role. Recruitment relied on the patient to explain the study; if this had not been done effectively, then informal carers may not participate. Patients may be reluctant to ask informal carers to participate if they feel that that the individual is already doing too much for them and participation may be seen as another burden.

## 8 CONCLUSIONS

This study establishes the importance of caring dyads as an analytical tool to better reveal the experience of illness than simply looking separately at patients with long‐term conditions and informal carers. Analysing the dyad as a unit, using separate interviews, enriches the perception of both the phenomenon and the shared experience of the phenomenon under study. This method of analysis holds much promise for deepening and broadening knowledge of the experience and management of illness.

Unique to this analytical approach is comparing and contrasting individual accounts of illness with the dyad. The dyadic view led to the generation of unique themes and subthemes that would otherwise not have been visible. In particular, we demonstrate that the shared experience of a phenomenon generates similar and contrasting experiences that impact on the management of illness and living with a complex medical condition.

## CONFLICT OF INTERESTS

The authors declare that there are no conflict of interests.

## Data Availability

Data are available on request due to privacy/ethical restrictions.

## APPENDIX 1 TOPIC GUIDE FOR INTERVIEW WITH PATIENTS

(1) **Introduction to interview**
  - Thank you for participating.
  - Introduction of lead researcher and others involved in the research (Aston University and LPT NHS Trust).
  - Overview of research and purpose.
  - Approximate duration of the interview is 45–60 min.
  - Proposed structure of interview—interested in what is important to them; no right or wrong answers, it is their perspective that we are interested in.
  - Consent to participate and approval to audio record the interview.
  - Confidentiality, anonymity of interview data and right to withdraw at any time.
  - Collection of basic demographic data and clinical data using the form at the end of this topic guide.
(2) **Questions**
  - Introduction (Note: The aim here is to get an idea of the person behind the story. Focus on the ‘now’—their current life. Also, to ease the person into the interview. Ask prompting questions to suit the person).
    – Could you tell me a little about yourself—how would you describe yourself based on your current life? Prompts:—What do you do (as in work, keeping themselves occupied etc.)?—What are your interests and hobbies?—Family/community support and social networks
    – How is your health at the moment?
  - What does health and well‐being mean to you?
    *(Idea of both physical health/well‐being and mental health within this)*
    – How would you describe your physical health/well‐being at the moment?
    – Have you got any concerns about your physical health/well‐being?
    – Tell me about the care you are getting for your physical health issue (use named example).
    – What things help your physical health? What things don't help your physical health?
  - Role of the pharmacist or pharmacy *(Explore both community and hospital pharmacy/pharmacist)*
    – How often do you visit a pharmacy or speak to a member of the pharmacy team?
    – What are the main reasons for visiting the pharmacy/speaking to the pharmacist?
    – Does your pharmacy/pharmacist help you with your medicines?
    – Does your pharmacy/pharmacist support your physical health/well‐being? Are any of these for health promotion or risk reduction (name example as appropriate e.g., diet, smoking cessation)?
    – Tell me about a time when the pharmacist has helped or given you advice about your physical health/well‐being.
    – What help or support would you like your pharmacist/pharmacy to provide?
    – What kind of relationship would you say you have with your pharmacist/pharmacy team?
  - Potential barriers
    – What things get in the way of you developing/improving your physical health/well‐being?
    – How do you think these might be overcome?
    – What do you think might get in the way of pharmacy/pharmacists supporting your physical health?
    – How do you think these might be overcome?
  - Facilitators/enablers
    – What things help you to develop/improve your physical health/well‐being?
    – How do they help you?
    – What would you like to make it easier to for you to do things for your physical health/well‐being?
    – What would make it easier for you to get support from your pharmacy/pharmacist?
    – What would make it easier for pharmacies/pharmacist give you support for your physical health?
  - Conclusions
    – Are there any other things you would like to add to these discussions?
    – Thanks for taking part in this study and for your time.

____________________________________

Please note that this document is for the Lead Researcher and their research team only and will not be given to the participants.

## APPENDIX 2 TOPIC GUIDE FOR INTERVIEW WITH INFORMAL CARERS

(3) **Introduction to interview**
  - Thank you for participating.
  - Introduction of lead researcher and others involved in the research (Aston University and LPT NHS Trust).
  - Overview of research and purpose.
  - Approximate duration of the interview is 45–60 min.
  - Proposed structure of interview—interested in what is important to them; no right or wrong answers, it is their perspective that we are interested in.
  - Consent to participate and approval to audio record the interview.
  - Confidentiality, anonymity of interview data and right to withdraw at any time.
  - Collection of basic demographic data
(4) **Questions**
  - Introduction (Note: The aim here is to get an idea of the person behind the story. Focus on the ‘now’—their current life. Ask prompting questions to suit the person).
    – Could you tell me a little about yourself—Prompts:—What do you do (as in work, keeping themselves occupied etc.)?—What are your interests and hobbies?—Family and social networks?
  - Considering the person, you care for and the support you give them: *(Idea of both physical health/well‐being and mental health within this)*
    – How would you describe the physical health/well‐being of the person you care for at the moment?
    – Have you got any concerns about their physical health/well‐being?
    – Tell me about the care they are getting for their physical health issue (used named example).
    – What things help the physical health of the person you care for? What things don't help?
  - Role of the pharmacist or pharmacy *(Explore both community and hospital pharmacy/pharmacist in relation to the person they care for)*
    – How often to do you visit a pharmacy or speak to a member of the pharmacy team?
    – What are the main reasons for visiting the pharmacy/speaking to the pharmacist?
    – Does your/the pharmacy/pharmacist help you with the medicines of the person you care for?
    – Does your/the pharmacy/pharmacist support their physical health/well‐being? Are any of these for health promotion or risk reduction (name example as appropriate e.g., diet, smoking cessation)?
    – Tell me about a time when the pharmacist has helped or given you advice about the physical health/well‐being of the person you care for.
    – What help or support would you like the pharmacist/pharmacy to provide?
    – What kind of relationship would you say you have with your pharmacist/pharmacy team?
  - Potential barriers
    – What things get in the way of the person you care for developing/improving their physical health/well‐being?
    – How do you think these might be overcome?
    – What do you think might get in the way of pharmacy/pharmacists supporting the physical health of the person you care for?
    – How do you think these might be overcome?
  - Facilitators/enablers
    – What things help develop/improve the physical health/well‐being of the person you care for?
    – How do they help?
    – What would you like to make it easier for the person you care for to do things for their physical health/well‐being?
    – What would make it easier for the person you care for get support from the pharmacy/pharmacist?
    – What would make it easier for pharmacies/pharmacist give the person you care for support for their physical health?
  - Conclusions
    – Are there any other things you would like to add to these discussions?
    – Thanks for taking part in this study and for your time.

____________________________________

Please note that this document is for the Lead Researcher and their research team only and will not be given to the participants.
